# Whole Genome Sequencing for Public Health Surveillance of Shiga Toxin-Producing *Escherichia coli* Other than Serogroup O157

**DOI:** 10.3389/fmicb.2016.00258

**Published:** 2016-03-03

**Authors:** Marie A. Chattaway, Timothy J. Dallman, Amy Gentle, Michael J. Wright, Sophie E. Long, Philip M. Ashton, Neil T. Perry, Claire Jenkins

**Affiliations:** Gastrointestinal Bacteria Reference Unit, Public Health EnglandLondon, UK

**Keywords:** whole genome sequencing, Shiga Toxin-producing *Escherichia coli*, serotyping, stx subtyping

## Abstract

Shiga toxin-producing *Escherichia coli* (STEC) are considered to be a significant threat to public health due to the severity of gastrointestinal symptoms associated with human infection. In England STEC O157 is the most commonly detected STEC serogroup, however, the implementation of PCR at local hospital laboratories has resulted in an increase in the detection of non-O157 STEC. The aim of this study was to evaluate the use of whole genome sequencing (WGS) for routine public health surveillance of non-O157 STEC by comparing this approach to phenotypic serotyping and PCR for subtyping the stx-encoding genes. Of the 102 isolates where phenotypic and genotypic serotyping could be compared, 98 gave fully concordant results. The most common non-O157 STEC serogroups detected were O146 (22) and O26 (18). All but one of the 38 isolates that could not be phenotypically serotyped (designated O unidentifiable or O rough) were serotyped using the WGS data. Of the 73 isolates where a flagella type was available by traditional phenotypic typing, all results matched the H-type derived from the WGS data. Of the 140 sequenced non-O157 isolates, 52 (37.1%) harboured *stx1* only, 42 (30.0%) had *stx2* only, 46 (32.9%) carried *stx1* and *stx2*. Of these, stx subtyping PCR results were available for 131 isolates and 121 of these had concordant results with the stx subtype derived from the WGS data. Of the 10 discordant results, non-specific primer binding during PCR amplification, due to the similarity of the stx2 subtype gene sequences was the most likely cause. The results of this study showed WGS provided a reliable and robust one-step process for characterization of STEC. Deriving the full serotype from WGS data in real time has enabled us to report a higher level of strain discrimination while stx subtyping provides data on the pathogenic potential of each isolate, enabling us to predict clinical outcome of each case and to monitor the emergence of hyper-virulent strains.

## Introduction

Shiga toxin-producing *Escherichia coli* (STEC) are considered to be a significant threat to public health due to the severity of gastrointestinal symptoms associated with human infection and the risk of cases developing Haemolytic Uraemic Syndrome (HUS; Byrne et al., 2015). STEC are zoonotic; transmission occurs by direct contact with animals or their environment, or by consumption of contaminated food or water (Byrne et al., 2014). The infectious dose is low (<10 organisms) and person-to-person spread is common, particularly in nursery school settings and in households with young children (Byrne et al., 2015).

In England, the current Standards for Microbiology Investigations protocols are specific for the isolation of non-sorbitol fermenting colonies of *E. coli* serogroup O157 on cefixime tellurite sorbitol MacConkey (CT-SMAC) agar. STEC serogroups other than O157 (non-O157 STEC) are not detected using this method (Byrne et al., 2014). However, since 2012 the implementation of commercial PCR assays for the detection of STEC in faecal specimens from cases with symptoms of gastrointestinal infection, at a twelve local hospital laboratories, has resulted in an increase in the detection of non-O157 STEC (Byrne et al., 2014).

Faecal specimens that are PCR positive for the Shiga Toxin (stx) genes at the local hospital laboratories in England are sent to the Gastrointestinal Bacterial Reference Unit (GBRU) at Public Health England (PHE) for isolation of STEC (Jenkins et al., 2012) and subsequent serotyping (Gross and Rowe, 1985). Recent advances in whole genome sequencing (WGS) have led to the development of a method for high throughput sequencing of bacterial genomes at low cost (Joensen et al., 2014). During 2014, we evaluated the use of WGS for routine public health surveillance of non-O157 STEC by comparing this approach to phenotypic serotyping and PCR for subtyping the stx-encoding genes (Persson et al., 2007).

## Materials and methods

All 167 strains of non-O157 STEC isolated during 2014 were phenotypically serotyped by the agglutination of antibodies raised in rabbits to the lipopolysaccharide O antigen and to the flagella H antigen (Gross and Rowe, 1985). Real-time PCR targeting *stx1* and *stx2* and the stx subtyping PCR was performed as previously described (Persson et al., 2007; Jenkins et al., 2012).

Genomic DNA extracted from 140 of the 167 strains of non-O157 STEC was fragmented and tagged for multiplexing with Nextera XT DNA Sample Preparation Kits (Illumina) and sequenced using the Illumina HiSeq 2500. A reference database, *SerotypeFinder*, containing the gene sequences encoding the 180 O antigen groups (*wzx, wzy, wzm*, and *wzt*) and the 53 H antigens (*fliC, flkA, fllA, flmA*, and *flnA*) was constructed and developed by Joensen et al. (2015). Using the *GeneFinder* tool (Doumith unpublished), FASTQ reads were mapped to the genes in the *SerotypeFinder* database using Bowtie 2 (Langmead and Salzberg, 2012) and the best match to each of the O and H determinants was reported with metrics including coverage, depth, mixture and homology in an XML format for quality assessment. Only *in silico* predictions of serotype that matched to a gene determinant at >80% nucelotide identity over >80% length were accepted. Stx subtyping was performed as described by Ashton et al. (2015). FASTQ sequences were deposited in the National Center for Biotechnology Information Short Read Archive under the bioproject PRJNA248064.

## Results

Whole genome sequences were available for 140 of the 167 non-STEC isolates reported in 2014 (Supplementary Table). Of these, 102 had a phenotypically derived serogroup, 25 did not agglutinate with the antisera in the serotyping scheme raised to the known *E. coli* serogroups and were designated “O unidentifiable,” and 13 did not express the O antigen and were designated “O rough.” Of the 102 isolates where phenotypic and genotypic serotyping could be compared, 98 gave fully concordant results (Supplementary Table). The most common non-O157 STEC serogroups detected were O146 (22) and O26 (18). There were 15 strains of STEC O55, all from cases linked to an outbreak in the South of England.

Of the four results that were not fully concordant, two isolates serogrouped as O186 phenotypically but were designated O123/O186 by *in silico* serotyping and one typed as O178 phenotypically and was designated O153/178 using WGS data. There was one mismatch; STEC O74 was identified as STEC O187 when the serotype was derived from the genome. The *in silico* serotyping method failed to type one isolate, STEC O146:H21, due to the short read sequences having low mapping coverage of the O antigen encoding genes. All but one of the 38 isolates that could not be phenotypically serotyped (designated O unidentifiable or O rough) were serotyped using the WGS data (Supplementary Table). The most common WGS derived serotypes that were untypable using the phenotypic approach were O91:H14, O117:H7, and O80:H2.

There were 102 isolates that were processed for H-typing, of which 29 were found to be non-motile and could not be typed. Of the 73 isolates where a flagella type was available by traditional phenotypic typing, all results matched the H-type derived from the WGS data. All the non-motile isolates were typable using the *in silico* serotyped by *in silico* serotyping (Supplementary Table).

Of the 140 sequenced non-O157 isolates, 52 (37.1%) harboured *stx1* only, 42 (30.0%) had *stx2* only, 46 (32.9%) carried *stx1* and *stx2*. Of these, stx subtyping PCR results were available for 131 isolates and 121 of these had concordant results with the stx subtype derived from the WGS data (Supplementary Table).

The most frequently detected stx subtype profile was stx1a only (38), most commonly associated with serotypes O103:H2 (14), O26:H11 (8), and O117:H7 (8) (Supplementary Table). There were 14 isolates with stx1c only, including O146:H21 (5) and O76:H19 (3). All stx1a and stx2b strains belonged to STEC O91:H14 (9). There were 23 isolates that had stx1c and stx2b associated with serogroups O146:H21 (9), O128:H2 (8), O113:H4 (2), and O174:H8 (2). The combination of stx1a and stx2a was found almost exclusively in O26:H11 (7), the one exception being an isolate of STEC O71:H2. Of the 21 isolates harbouring *stx2a* only, 15 were STEC O55:H7 and two were O26:H11. Of the 10 isolates that had *stx2d*, three belonged to O146:H21 (and also had *stx1c*), six were O80:H2 and one was O26:H11. Other rare subtypes (one case of each) included stx2e (O8:H9) and stx2g (O187:H28) (Supplementary Table).

There were 10 cases of HUS in 2014 (including five cases belonging to an outbreak of STEC O55:H7), eight had STEC harbouring *stx2a*, one had STEC O80:H2 carrying *stx2d*, and O103:H2 stx1a was isolated from the tenth case.

## Discussion

The results of this study showed WGS provided a reliable and robust one-step process for characterisation of STEC. Previous studies have shown an increasing number of strains of STEC reported as “O group unidentifiable” due to antisera failing quality control procedures, unresolvable cross reactions, lack of expression of O antigens (designated “rough”) or novel serogroups (Jenkins et al., 2003; Byrne et al., 2014). In this study, all but one of the isolates that were previously phenotypically untypable, were serotyped using data derived from the genome.

Of the 10 mismatched results identified in the comparison between the stx subtyping PCR (Scheutz et al., 2012) and the WGS approach (Ashton et al., 2015), all 10 had additional stx subtypes detected by PCR that were not identified in the WGS data (Supplementary Table). Non-specific primer binding during PCR amplification, due to the similarity of the stx2 subtype gene sequences was the most likely cause.

Historically, the most common stx profile of STEC O26 was stx1a but over the last 10 years a more virulent STEC O26 variant harbouring *stx2a* has emerged across Europe (Bielaszewska et al., 2013). The enhanced strain characterisation data that WGS provides facilitated the surveillance of emerging strains of STEC associated with more severe disease (for example STEC O55:H7 stx2a, STEC O26:H11 stx2a, STEC O80:H2 stx2d) and with novel stx profiles (for example STEC O26:H11 stx2d) and enabled us to compare data with colleagues in the field (Mariani-Kurkdjian et al., 2014; Delannoy et al., 2015). Previous studies have also reported an association between stx2a and severe disease (Ethelberg et al., 2004; Byrne et al., 2014). The *eae* gene was detected in 62 (44%) of the 140 non-O157 STEC isolates. All 10 isolates from the HUS cases had *eae*. None of the STEC strains in this data set had *aggR*, previously detected in highly pathogenic STEC variants (Boisen et al., 2015).

Prior to the implementation of WGS, due to limited resources and time constraints, H-typing and stx subtyping of STEC were not routinely reported by GBRU. Deriving the full serotype from WGS data in real time has enabled us to report a higher level of strain discrimination while stx subtyping provides data on the pathogenic potential of each isolate, enabling us to predict clinical outcome of each case and to monitor the emergence of hyper-virulent strains.

## Author contributions

MW and NP isolated the STEC and performed the real-time stx PCR. AG and SL performed the phenotypic serotyping, stx subtyping PCR and extracted the DNA. CJ, MC, MW, and NP implemented the wet lab WGS pipelines and performed analysis. TD and PA implemented the bioinformatics pipelines and performed analysis. CJ, MC, and TD wrote the manuscript.

## Funding

This work was supported by the National Institute for Health Research and Health Protection Research Unit in Gastrointestinal Infections at the University of Liverpool.

### Conflict of interest statement

The authors declare that the research was conducted in the absence of any commercial or financial relationships that could be construed as a potential conflict of interest.
